# Dr. Falconer, on the Portland Powder

**Published:** 1801-05

**Authors:** William Falconer

**Affiliations:** Bath


### *To the Editors of the Medical and Phyfical Journal.*

GENTLEMEN,

A Printed paper was put into my hands a few days ago, fubfcribed D. WHITEHEAD, No. 16, Charlotte-ftreet, Bloomsbury, recommending a revival of the old remedy for the Gout, known by the name of the Portland Powder, which is here faid to be improved, and is exhibited in the form of an expenfive and lucrative noftrum.

As the effects of this preparation have long been recognized by profeffional perfons as injurious and mifchievous, and as the authorities cited in the paper which recommends it, are much mifreprefented and perverted, I wifh to lay before the public what I apprehend to be the true ftate of the cafe, and to caution mankind againft the trial of a remedy at once fo deceitful and fo dangerous. From what is faid in the paper above alluded to, we fhould be led to believe that this remedy was purchafed and difperfed by the prefent* Duke of Portland; whereas, it was by his father, many years ago. The prefent nobleman owes his amendment, and indeed his recovery from this painful complaint, to a meritorious and fteady adherence to an abftemious and regular courfe of diet, which confifts nearly of vegetable fubftances; the mixture of animal food being very fmall, and that of the mildeft kind: to this is joined a total abftinence from all fermented liquors; and it is to this judicious management, and not to any medicine, either regularly prefcribed or empirically recommended, that he afcribes his freedom from this hereditary malady. The powder which the late duke took himfelf, and of which he directed copies of the compofition, and the manner of its preparation, to be given gratuitoufly to all who defired it, is as follows:

* "His Grace the Duke of Portland, who had, I believe, been perfonally an extreme fufferer from the Gout, became acquainted with a medicine in Switzerland, for the cure of that inveterate diforder; and after the moft indubitable evidence of its intrinfic worth, purchafed the receipt for the benefit of his country. This medicine, in a highly improved form, I beg leave to introduce to your notice; and hope, that the fanction of the illuftrious family by whom the receipt was firft obtained, will fecure an unbiaffed attention to the neceffary improvements now introduced into its compofition." Mr. WHITEHEAD's Hand-Bill, page 1.

"For the GOUT or RHEUMATISM.

"R. Ariſtolochia rotunda (or birthwort), gentian, root; Germander, ground pine, centaury, tops and leaves.

"Take of all theſe, well dried, powdered and ſifted as fine as you can, equal weight; mix them well together, and take one drachm of this mixed powder every morning faſting, in a cup of wine and water, broth, tea, or any other vehicle you like beſt; keep faſting an hour and half after it. Continue this for three months without interruption; then diminiſh the doſe to three-fourths of a drachm for three months longer; then to half a drachm for ſix months more, taking it regularly every morning if poſſible. After the firſt year, it will be ſufficient to take half a drachm every other day. As this medicine operates inſenſibly, it will perhaps take two years before you receive any great benefit, ſo you muſt not be diſcouraged, though you do not perceive at firſt any great amendment; it works ſlow, but ſure; it doth not confine the patient to any particular diet, ſo one lives ſoberly, and abſtains from thoſe meats and liquors that have always been accounted pernicious in the Gout, as Champaigne, drams, high ſauces, &c.

"N. B. In the Rheumatiſm that is only accidental, and not habitual, a few of the drachm doſes may do; but if habitual, or of long duration, then you muſt take it as for the Gout; — the remedy requires patience, as it operates but ſlow in both diſtempers."

The ingenious and learned Dr. JOHN CLEPHANE has given an excellent account of this very ancient preparation, in the firſt volume of the Medical Obſervations and Inquiries. It is mentioned, he obſerves, with very little variation from the above receipt, by Galen in the ſecond century; by Cælius Aurelianus, (from Soranus) who lived about the ſame time; by Aëtius and Alexander Trallianus in the fifth century; by Paulus Ægineta in the ſeventh century; by Myrepſus in the twelfth; by Franciſcus de Pedemontio, A.D. 1400; by the Prince of Mirandola, about 1480; by Tournefort in later times; and at a period ſtill later, it was transferred into the Paris Pharmacopœia, under the title of Pulvis Arthriticus Amarus.

This powder was given in the doſe of about a drachm, daily, for a year; as many of thoſe remedies called antidoti* were, and theſe directions are nearly copied in thoſe given for the uſe of the Portland powder, ſave that the latter is directed to be perſiſted in for a longer time.

But

* They were called from thence Annalia Medicamenta. Cæl. Aurel.

But though it cannot be denied that the ancient writers recommended, in fome cafes, thefe bitter preparations as remedies for the Gout, yet they advifed them with confiderable referve, and an apprehenfion of their danger.

Soranus,* who advifed them, cautions againft their being long continued; as, he fays, they brought on fome perfons acute complaints; on others, apoplexy; on others, pleurify and peripneumony; and in fome cafes, difficulty of breathing, or dyfpnœa.

All of the writers on the fubject, caution againft the indifcriminate ufe of it in all cafes and habits, as they affure us that they are extremely hurtful in hot and bilious habits, and proper only in cold phlegmatic conftitutions. They alfo judged them to be very dangerous in cafes of long ftanding, and advife no trial of them to be made where the complaint has exifted *five*, or at moft, *seven* years.

Such is the abftract of the accounts given of this remedy by the writers of antiquity. Let us now turn to the modern accounts, and particularly to that of the celebrated Dr. Cullen, who is vouched as evidence of the fact by Mr. Whitehead; wherein it will appear, with what impropriety, and under what mifreprefentation, this admirable phyfician has been introduced as encouraging a practice he always reprobated in his converfation, as I can teftify, and in his writings, which are open to the perufal of every one.

" In every inftance," fays Dr. Cullen, in his †Practice of Phyfic, " which I have known of its exhibition for the length of time prefcribed, the perfons who had taken it were, indeed, afterwards free from any inflammatory affection of the joints; but they were afterwards affected with many fymptoms of the atonic Gout, and *all*, foon after finifhing their courfe of the medicine, have been attacked with apoplexy, afthma, or dropfy, which proved fatal."

In a later publication of the fame eminent writer, he obferves, that " the effects of this powder in modern times, have been very much on the fame footing with the ancient. It is poffible, fays he, that feveral perfons may have taken the Portland powder, and other bitters, with feeming great advantage; but I have not had opportunity to know the fequel of the whole of fuch perfons lives, fo as to fay pofitively how far, in any cafe, the cure continued fteady for a life of fome years after, or what accidents happened to their health.

* Cælii. Aurel. L. v. c. 2.

"But I have had occaſion to know, or to be exactly informed, of the fate of nine or ten perſons who had taken this medicine for the time preſcribed, which is two years. Theſe perſons had been liable for ſome years before to have a fit of regular or very painful inflammatory gout, once at leaſt, and frequently twice in the courſe of a year; but after they had taken the medicine for ſome time, they were quite free from any fit of inflammatory gout, and particularly when they had completed the courſe preſcribed, had never a regular fit, or any inflammation of the extremities, for the reſt of their life.

"In no inſtance however that I have known, was the health of theſe perſons tolerably entire. Soon after finiſhing the courſe of their medicine, they became valetudinary in different ſhapes, and particularly were much afflicted with dyſpeptic and what are called nervous complaints, with lowneſs of ſpirits. *In every one of them*, before a year had paſſed after finiſhing the courſe of the powders, ſome hydropic ſymptoms appeared, which gradually increaſing in the form of an aſcites or hydrothorax, eſpecially the latter, joined with anaſarca, in leſs than two or at moſt three years proved fatal.

"Theſe accidents happening to perſons of ſome rank, became very generally known in this country, and has prevented all ſuch experiments ſince." Such are the words of Dr. Cullen; and the reader will, I am certain, join with me in cenſuring the diſingenuous perverſion of them in the printed paper alluded to*. Had the whole of the paſſages I have cited been inſerted into Mr. Whitehead's recommendation of the remedy, who could have imagined Dr. Cullen could be introduced as bearing teſtimony in favour of its uſe? But the real opinion of Dr. Cullen is ſuppreſſed, and only ſo much of the effects of the powder is inſerted on his authority, as may ſerve the purpoſe of perſuading thoſe who have not had an opportunity of knowing his real ſentiments. In juſtice to him, and to mankind, I now lay them before the public, and I am confident the candid and benevolent part of the world will think me fully juſtified in publiſhing this caution in the uſe of a remedy of this character. But its ill effects were not known to our own countrymen only; Werlhoff, a German practitioner of eminence, and firſt phyſician to his late Majeſty for the Electorate of Hanover, agrees in condemning theſe bitter remedies

* "This celebrated remedy, ſince its introduction into England, is acknowledged by the moſt eminent of the faculty to be capable of removing the paroxyſms of the gout; and we may freely conclude the teſtimony of the celebrated Dr. Cullen, of Edinburgh, undeniable evidence of the fact."

Mr. WHITEHEAD's Advertiſement, or Hand-bill, Page 1.

remedies for the gout. After faying that the return of the painful paroxyfms is thereby prevented, he adds, " that by the exceffive ufe of thefe bitter remedies, he has known the digeftive power of the ftomach to be fo weakened, as to produce a lofs of appetite, and proper concoction of the food, which has accelerated the death, inftead of reftoring the health of thofe who had ufed them, who thus paid the fevere penalty attendant on the trial of thefe unlucky and mifchievous remedies."*

Murray, the Gottingen profeffor, gives in his Apparatus Medicamentum, a fimilar account; and adds, " that the powder produced in many inftances, apoplexy, palfy, and acute diforders, together with difficulty of breathing, a dry cough, and tubercles of the lungs, which proved fuddenly mortal.†

The reputation of this medicine having declined before I had any opportunity of obferving its effects at the time of taking, and its mifchievous confequences having prevented its having many living vouchers of its fuccefs, I cannot fay any thing of it from my own experience. I remember, indeed, one perfon far advanced in years, who was I believe a Proctor in the Ecclefiaftical Court at York, who was pointed out to me as a remarkable inftance of one who had furvived the effects of this remedy. He appeared in good health, and had not I believe experienced any ill effects from the powder. But this is, as far as my information goes, a folitary inftance, and no more to be depended on as an encouragement to the trial of the remedy than an extraordinary cafe of excefs in fpirituous liquors, which ftill did not appear to abridge life, or injure health, would be to encourage the indulging in that odious and poifonous beverage.

*Bath, Feb.* 12, 1801. WILLIAM FALCONER.

---

* Sed ex nimio horum amaricantium ufu, fermentum ftomachi adeo debilitatum effe memini, ut nonnuli appetitum amiserint, cibos non concoxerint, mortem hinc potius, quam fanitatem accelerarint; malique ex infaufti remedii fævas dederint pœnas. WERLHOFF, Caut. Medicæ, Page 346.

† Ex pulvere arthritico multi apoplexiam, paralyfin, vel morbos acutos, fenes præcipue, contraxerunt. Et in homine quodam, arthritis quidem inde fedata, fed refspiratio difficilis, tuffis ficca, morsque fubitanea fucceffit, tuberculis pulmonum poft mortem confpicuis.

